# A‐to‐I RNA Editing in *Klebsiella pneumoniae* Regulates Quorum Sensing and Affects Cell Growth and Virulence

**DOI:** 10.1002/advs.202206056

**Published:** 2023-04-21

**Authors:** Xin‐Zhuang Yang, Tian‐Shu Sun, Pei‐Yao Jia, Sheng‐Jie Li, Xiao‐Gang Li, Yanan Shi, Xue Li, Haotian Gao, Huabing Yin, Xin‐Miao Jia, Qiwen Yang

**Affiliations:** ^1^ Department of Clinical Laboratory State Key Laboratory of Complex Severe and Rare Diseases Peking Union Medical College Hospital Chinese Academy of Medical Sciences and Peking Union Medical College Beijing 100730 China; ^2^ Medical Research Center State Key Laboratory of Complex Severe and Rare Diseases Peking Union Medical College Hospital Chinese Academy of Medical Sciences and Peking Union Medical College Beijing 100730 China; ^3^ School of Engineering University of Glasgow Glasgow G12 8QQ UK

**Keywords:** badR, cell growth, *Klebsiella pneumoniae*, RNA editing regulation, virulence

## Abstract

Millions of adenosine (A) to inosine (I) RNA editing events are reported and well‐studied in eukaryotes; however, many features and functions remain unclear in prokaryotes. By combining PacBio Sequel, Illumina whole‐genome sequencing, and RNA Sequencing data of two *Klebsiella pneumoniae* strains with different virulence, a total of 13 RNA editing events are identified. The RNA editing event of *badR* is focused, which shows a significant difference in editing levels in the two *K. pneumoniae* strains and is predicted to be a transcription factor. A hard‐coded Cys is mutated on DNA to simulate the effect of complete editing of *badR*. Transcriptome analysis identifies the cellular quorum sensing (QS) pathway as the most dramatic change, demonstrating the dynamic regulation of RNA editing on *badR* related to coordinated collective behavior. Indeed, a significant difference in autoinducer 2 activity and cell growth is detected when the cells reach the stationary phase. Additionally, the mutant strain shows significantly lower virulence than the WT strain in the *Galleria mellonella* infection model. Furthermore, RNA editing regulation of *badR* is highly conserved across *K. pneumoniae* strains. Overall, this work provides new insights into posttranscriptional regulation in bacteria.

## Graphical abstract

Through the analysis of RNA editing regulation in *Klebsiella pneumonia*, it is reported that adenosine to inosine RNA editing on *badR* gene regulates quorum sensing and has a certain regulatory effect on the growth and virulence of bacteria. The conclusion suggests that in addition to the mutation of the genome, it is necessary to consider posttranscriptional regulation in future research on bacteria.

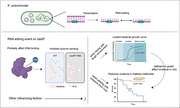

## 1 Introduction

RNA editing is a posttranscriptional process that introduces differences between RNA and its corresponding DNA template.^[^ ^1^ ^]^ With the advent of next‐generation sequencing, millions of RNA editing sites have been identified in humans.^[^ ^2^, ^3^ ^]^ The most prevalent type of RNA editing in metazoans is the conversion of adenosine to inosine (A‐to‐I) catalyzed by adenosine deaminase acting on RNA (ADAR) enzymes.^[^ ^4^, ^5^, ^6^ ^]^ Recent studies have provided functional evidence for a series of editing events such as events in coding regions recoding amino acid messages, events located near exon‐intron boundaries modulating splicing strength, and some intergenic editing events implicated in the biogenesis and targeting of microRNAs and piwi‐interacting RNAs.^[^ ^7^, ^8^ ^]^ An increasing amount of work has also led to the discovery and identification of RNA editing sites in fungi.^[^ ^9^, ^10^, ^11^ ^]^ However, RNA editing regulation in bacteria has rarely been reported. Recently, Bar‐Yaacov et al. provided evidence of RNA editing in *Escherichia coli* on *hokB*, which encodes a toxin that confers antibiotic tolerance, that increases as a function of cell density and enhances the toxicity of the protein.^[^ ^12^ ^]^ Furthermore, the authors found that tRNA‐specific adenosine deaminase (TadA) was also identified as the catalyzing enzyme of A‐to‐I editing on mRNA. In addition, Nie et al. identified A‐to‐I editing events of *fliC*, and they discovered that the S128P editing event is induced by H_2_O_2_ and demonstrated its importance in bacterial pathogenicity and adaptation to oxidative stress.^[^ ^13^ ^]^ These studies reported mRNA editing events in bacteria and proved the important function of RNA editing regulation. It is still unclear whether bacterial growth, virulence, or stress responses are regulated by RNA editing in other bacterial strains such as *Klebsiella pneumoniae (K. pneumoniae)*.

*K. pneumoniae*, an important pathogen, can cause a wide range of infections including pneumonia, urinary tract infection, bloodstream infection, and liver abscess.^[^ ^14^, ^15^, ^16^ ^]^ Moreover, some *K. pneumoniae* strains have various virulence factors, such as *rmpA*, *rmpA2*, *iucABCD*, and *iroBCDN*, which confer hypervirulence. This hypervirulent *K. pneumoniae* leads to more serious infections and higher mortality rates. However, there were also some strains that had a highly virulent phenotype but no hypervirulence genes and the reason for this remains unclear.^[^ ^17^, ^18^ ^]^ To date, most studies on the virulence of *K. pneumoniae* have focused on the DNA level, and there are few reports on the transcription level. In this study, we developed a strict pipeline for RNA editing identification in bacteria by using PacBio genome sequencing data, Illumina whole genome sequencing (WGS) data, and strand‐specific RNA Sequencing (RNA‐Seq) data. By applying this pipeline, we profiled and identified RNA editing events in one hypervirulent isolate and one classic strain of *K. pneumoniae*. Specific editing events were identified and quantified between the two strains, and their regulatory effects on bacterial growth and virulence were demonstrated.

## 2 Results

### 2.1 Identification of RNA Editing Events in *K. pneumoniae*

**Figure 1:**
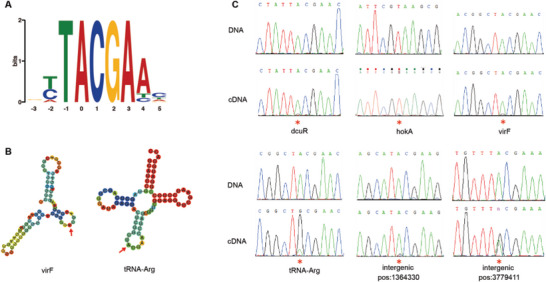
A) All RNA editing sites share a common DNA motif, in that the nucleotide 5′ to the editing site significantly favored T, while the 3′ nucleotides favored CGA. The center 0 base indicates the editing sites. B) RNA secondary structure modeling predicted that edited sites are embedded within a loop. Here, the secondary structure of *virF* (as well as tRNA‐Arg) is presented. C) Sanger validation of both DNA and RNA editing sites identified in the R16 strain. Editing sites are highlighted with red pentacles.

To identify RNA editing events in *K. pneumoniae*, we developed a new strict pipeline that was a modified and improved version of our previous pipeline.^[^ ^19^, ^20^ ^]^ Taking advantage of the long reads of the PacBio third‐generation sequencing platform, we obtained the complete genome sequence of *K. pneumoniae* R16 (a hypervirulent strain) (more details and the refined genome structure have been published in our previous work).^[^ ^18^ ^]^ Then, WGS was performed by Illumina on the DNA of *K. pneumoniae* strain R16, and RNA‐Seq in strand‐specific mode was performed on the same strain. WGS and RNA‐Seq data, which have higher sequencing abundance and lower sequencing error rates, were mapped to the assembled reference genome, and stringent parameters were applied to identify RNA editing events that can manifest themselves as base differences between the DNA and RNA sequences. Making full use of the strand‐specific mode of RNA‐Seq, we separated reads into different transcriptional directions for more accurate identification of RNA editing events. In total, we found 13 RNA editing sites in *K. pneumoniae* R16. 12 of them were A‐to‐G, and 7 were within known ORFs (**Table** **1** and Table S1, Supporting Information). In addition, multiple features of these candidate sites indicated that they represent bona fide RNA editing events in bacteria. 1) Three RNA editing sites were identified on tRNA‐arg, which have been previously reported.^[^ ^12^, ^21^ ^]^ 2) All editing sites within ORFs were predicted to recode a tyrosine (Tyr) encoded by the TAC codon into a cysteine (Cys) encoded by the TGC codon, presenting stronger consistency than other bacterial species (Table 1).^[^ ^22^ ^]^ 3) All A‐to‐G editing events were embedded within a five‐base‐long motif TACGA, with the edited A at the second position (**Figure** **1**A). 4) Considering that TadA was reported as an editing enzyme for most editing events in *E. coli*,^[^ ^12^ ^]^ we further checked the RNA secondary structure of editing sites identified in our study, and they were also embedded within a loop in most of the sites that satisfied the structure recognized by TadA (Figure 1B and Figure S1, Supporting Information). Finally, eight of the A‐to‐G sites were successfully experimentally verified by Sanger sequencing of both DNA and the corresponding cDNA (Figure 1C). The other four sites failed, which might be due to a lower editing frequency (<0.05; Table 1), and one C‐to‐U mutation site was confirmed to be a negative result. The high validation rate suggested that the RNA editing sites identified in this study are verifiable.

**Table 1:** RNA editing events identified in *Klebsiella pneumoniae*

Position	Strand	Gene symbol	Relative position on gene	Editing format	Context nucleotide	AA change	Average editing level in R16 strain [%]	Average editing level in QD110 strain [%]	Editing level comparison (p‐value)
368 437	−	*dcuR*	398	A→G	TAC→TGC	Tyr→Cys	9.67	6.23	0.161
1 364 330	+	intergenic	‐	A→G	TAC→TGC	–	7.50	NAN	NAN
1 393 718	−	*sbmC*	311	A→G	TAC→TGC	Tyr→Cys	2.07	0.13	5.00E‐04
2 079 117	−	tRNA‐Arg	35	A→G	TAC→TGC	–	67.48	59.71	0.089
2 079 422	−	tRNA‐Arg	35	A→G	TAC→TGC	–	74.22	73.52	0.850
2 079 787	−	tRNA‐Arg	35	A→G	TAC→TGC	–	76.48	69.40	0.131
2 151 769	−	*hokA*	137	A→G	TAC→TGC	Tyr→Cys	35.09	12.60	0.085
2 255 195	+	*ddrA*	131	A→G	TAC→TGC	Tyr→Cys	NAN	3.66	NAN
3 429 483	+	*rep*	1450	A→G	TAC→TGC	Tyr→Cys	1.14	2.57	0.462
3 779 411	+	intergenic	–	A→G	TAC→TGC	–	65.20	43.11	9.68E‐04
3 969 107	+	*badR*	295	A→G	TAC→TGC	Tyr→Cys	4.34	9.24	0.021
4 939 002	+	*moaC*	379	A→G	TAC→TGC	Tyr→Cys	2	2.15	0.938
5 325 069	+	*virF*	91	A→G	TAC→TGC	Tyr→Cys	14.89	0	0.008

^*^NAN: Reads coverage of editing sites less than 5.

### 2.2 Comparison of RNA Editing Sites between Hypervirulent and Classic *K. pneumoniae*

**Figure 2:**
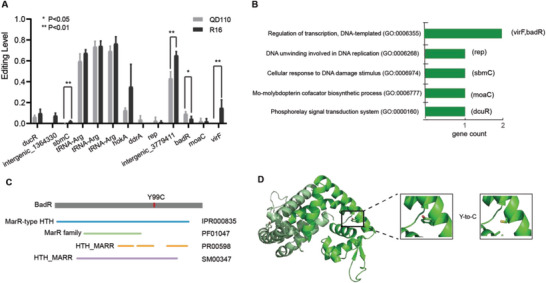
A) RNA editing levels measured by RNA‐Seq of two strains are presented (replicates = 4). Editing events with significantly different levels between the two strains were specifically labeled (*t*‐test). B) GO annotations for eight genes with editing sites. Two genes missing GO annotations are not shown. C) Predicted domains for BadR. The editing site that causes the Tyr‐to‐Cys transition at position 99 at the protein level is highlighted with a red line. D) Ribbon representation of BadR (modeled by SWISS‐MODEL) and close‐up views of Y99 and mutant C99.

To further explore whether RNA editing has a regulatory effect on the bacterial growth or virulence of *K. pneumoniae*, we analyzed the RNA editing events of a classic *K. pneumoniae* strain (QD110) and compared it with the hypervirulent strain (R16). By applying the RNA editing identification pipeline to the RNA sequencing data of QD110, 11 A‐to‐G RNA editing events were found, and 10 of them were also detected in strain R16 (Table 1 and Table S1, Supporting Information). By combining all RNA editing events identified in two *K. pneumoniae* strains, we observed that some presented significantly different editing levels between hypervirulent and classic strains (*t*‐test; **Figure** **2**A). Gene ontology (GO) annotation revealed that the biological functions of genes under RNA editing regulation were involved in the regulation of transcription, DNA replication, the Mo‐molybdopterin cofactor biosynthetic process, and the phosphorelay signal transduction system (Figure 2B). VirF is a primary regulator of plasmid‐encoded virulence genes and can activate the transcription of *virG* and *virB*, which are activators of the virulence regulon.^[^ ^23^, ^24^ ^]^ BadR was predicted to be a transcription factor belonging to the MarR (members of the multiple antibiotic resistance regulator) family, which is critical for bacterial cells to respond to chemical signals.^[^ ^25^ ^]^ HokA, belonging to the Hok family of host‐killing toxins, is the toxic component of a type I toxin‐antitoxin (TA) system. Interestingly, the function of RNA editing events on *hokB*, which is a homologous gene of *hokA*, was previously confirmed in *E. coli*.^[^ ^12^ ^]^ All the above results demonstrated that RNA editing regulation occurs on some key genes in *K. pneumoniae*, suggesting that it may play an important role. The differential RNA editing levels between the two strains also suggested that these editing events might have an impact on the differences in cell growth or virulence between the hypervirulent and classic *K. pneumoniae* strains.

### 2.3 RNA Editing of *badR* Causes Changes in Quorum Sensing and Affects the Growth and Virulence of *K. pneumoniae*

Among all the genes that underwent RNA editing events with significantly different editing levels between hypervirulent and classic *K. pneumoniae* strains (Figure 2A), we focused on *badR*, which was predicted to be a transcription factor and might have a global effect on bacteria. Considering that the biological function annotation of *badR* in bacteria is poor, we further analyzed the functional domain by entering the amino acid sequence of BadR into InterPro. Based on the results, BadR contains MarR‐type HTH domains (Figure 2C), which were reported to be DNA‐binding, winged helix‐turn‐helix (wHTH) domains present in transcription regulators of the MarR/SlyA family that are involved in the development of antibiotic resistance and virulence.^[^ ^26^, ^27^ ^]^ PROVEAN was then used to predict the impact of the Y99C (Tyr to Cys at position 99) substitution, induced by RNA editing, on the biological function of the BadR protein, and the result showed “Deleterious” with a PROVEAN score of −5.555. Furthermore, protein modeling was performed for Y99 and C99 separately to detect the influence of RNA editing on protein structure (Figure 2D). The result showed that BadR consists of a dimer, and the structure shows that *α*‐helices in the N‐ and C‐terminal regions of each monomer fold around and interdigitate with those of the other subunits to form a well‐packed hydrophobic core. The DNA‐binding lobe of each subunit could act independently and affect specificity. Movement of the lobes relative to each other would require distortion of the helices that link them to the N‐/C‐terminal domain, including the 94–117 (*α*5 helix). The variation in position 99 may help to accommodate relative shifts of the two DNA‐binding lobes of the dimer that might occur upon DNA‐binding; furthermore, this variation might have an influence on target preference.^[^ ^28^ ^]^

**Figure 3:**
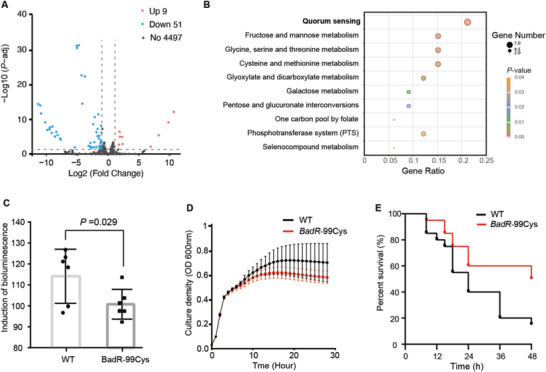
A) Differential gene expression between the WT and *badR*‐99Cys mutant strains. Red and blue dots indicate up‐ and downregulated genes, respectively. Grey dots indicate that the changes do not reach a significant level. B) KEGG enrichment analysis for differentially expressed genes. The top ten significant pathways are shown in order. C) AI‐2 production was detected using the bioluminescence of *Vibrio harveyi* strain BB170 at the 4th hour of incubation, and its activity of *badR‐*99Cys was lower than that of WT (*p*‐value = 0.029). Each dot represents an independent bacterial growth experiment. D) The cellular growth rates of WT and *badR*‐99Cys were examined 12 times by recording the optical density at 600 nm of cell cultures for 28 h. Each point represents the current cellular density each hour. *BadR*‐99Cys showed a lower growth rate after the 8th hour (*p*‐value = 0.002). E) The virulence of WT and *badR*‐99Cys was measured in the *Galleria mellonella* infection model with 15 larvae for each group. The ordinate represents the percentage of surviving individuals at each time point. The endpoint was the 48th hour of infection. *badR*‐99Cys showed a lower survival rate than WT after the 10th hour of infection (*p*‐value = 0.021).

As BadR is predicted to be a transcription regulator and Y99C might influence target selection, we further asked how the change can affect the transcriptome profile of *K. pneumoniae*. To answer this question, we mutated the genomic *badR* gene using the CRISPR/Cas9 system in the QD110 strain, with the codon TGC encoding a Cys hard‐coded into the DNA (*badR*‐99Cys) to mimic complete editing at this site (Figure S2, Supporting Information). Then, we performed whole‐transcriptome sequencing for the WT strain and the mutant strain *badR*‐99Cys. Differential gene expression analysis revealed that 9 genes were significantly upregulated and 51 genes were significantly downregulated in the mutant strain compared with the WT strain (**Figure** **3**A). Interestingly, the Kyoto Encyclopedia of Genes and Genomes (KEGG) enrichment analysis showed that the most enriched pathway of differentially expressed genes (DEGs) was quorum sensing (QS, *p*‐value = 0.0009, Figure 3B), as the Lsr family genes LsrA, LsrB, LsrC, LsrD, LsrF, LsrG, and LsrK, which are involved in autoinducer 2 (AI‐2) importation and phosphorylation,^[^ ^29^ ^]^ were downregulated in the mutant strain (Table S2, Supporting Information). To further examine the change in the QS system in the mutant strain, we detected AI‐2 activity using a reporter.^[^ ^30^ ^]^ To capture the moment of AI‐2 production by *K. pneumoniae*, we first dynamically examined cell‐free cultures incubated for ≈0–4 h. In our culture system, the production of AI‐2 was detected at the 4th hour (Figure S3, Supporting Information), while cell growth reached a plateau at this moment (Figure S4, Supporting Information). The AI‐2 activity in the mutant strain *badR*‐99Cys was significantly downregulated compared with that in the WT strain, suggesting that the effect of RNA editing on *badR*‐99Cys may manifest as cell density becomes saturated. (Figure 3C, *p*‐value = 0.029, *t*‐test). Moreover, the editing level of *badR* decreased almost tenfold as cells grew from the logarithmic phase to the stationary phase (Figure S4, Supporting Information). To examine whether this editing event affects the growth of *K. pneumoniae*, we compared the growth of the *K. pneumoniae* WT strain and the mutant strain *badR*‐99Cys. Indeed, a significant difference in maximum cellular density was detected when reaching the stationary phase (Figure 3D, *p*‐value = 0.002, Mann–Whitney test), indicating that the RNA editing of *badR* was detrimental to sustaining cells through the infertile condition. The ability of bacteria to withstand adversity is also an important factor in the survival of the host. In the *Galleria mellonella* infection model, the mutant strain *badR*‐99Cys showed lower virulence than the WT strain (Figure 3E, *p*‐value = 0.021, Mantel–Cox test).

Considering that bacteria are easily stimulated by external environmental factors, resulting in a stress response process, we examined the variation of RNA editing level of *badR* gene in *K. pneumoniae* with stimulated by high temperatures, high salinity, and oxidation, which are the most common situations bacteria deal with. The results showed that there was no significant change in RNA editing level at this site (Figure S5, Supporting Information, *t*‐test), which may suggest that the RNA editing event at this site might be not a transcriptional regulation that is widely activated by stress.

Beyond *badR*, the A‐to‐G editing site of position 5 325 069 on *virF* also had a significantly different RNA editing level between the two strains (Figure 2A). VirF is a primary regulator of plasmid‐encoded virulence genes and can activate the transcription of *icsA* (*virG*) and *virB*, which are activators of the virulence regulon.^[^ ^23^, ^24^ ^]^ Thus, we also mutated the genomic *virF* using the CRISPR/Cas9 system in the QD110 strain, with the codon TGC encoding a Cys hard‐coded into the DNA (*virF*‐31Cys). To examine the influence of this editing event on *K. pneumoniae*, we compared the growth of the WT strain and mutant strain, and no significant difference was detected (Figure S6A, Supporting Information, *p*‐value = 0.124, Mann–Whitney test). In the *G. mellonella* infection model, the mutant strain *virF*‐31Cys also showed similar virulence to that of the WT strain (Figure S6B, Supporting Information, *p*‐value = 0.545, Mantel–Cox test).

### 2.4 RNA Editing of *badR* was Highly Conserved across *K. pneumoniae* Strains

**Figure 4:**
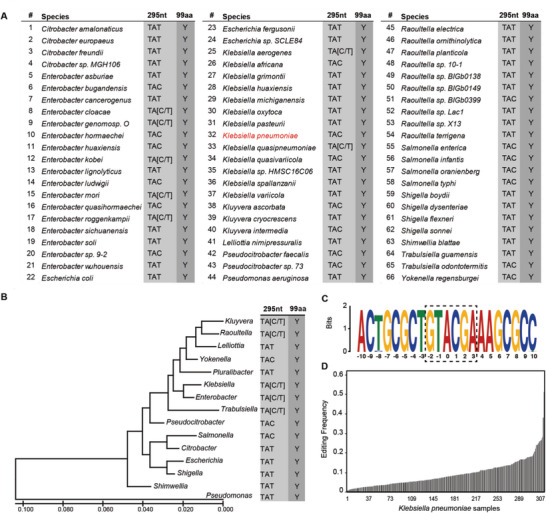
The conservation of the RNA editing site on *badR*. A) Multiple sequence alignment of BadR at position 99 from a representative nonredundant set of orthologs from bacterial species harboring the *badR* gene. B) A maximum likelihood phylogenetic tree based on the 16S rRNA gene showing the amino acid composition at BadR position 99 in each bacterial genus. C) Completely consistent sequence context surrounding the editing site on *badR* for all 74 strains of *Klebsiella pneumoniae*. The 0‐base indicates the editing position. D) RNA editing in *badR* was identified in publicly available *K. pneumoniae* (309) samples with sufficient coverage (≥10 reads) of RNA reads.

To further examine the conservation of RNA editing sites on *badR*, we performed multiple sequence alignment of BadR with a representative nonredundant set of orthologs from 66 bacterial species (764 strains), and we found the interplay between the TAT and TAC codons at position 99 (**Figure** **4**A,B). Notably, 63.9% (488/764) of strains had the codon TAT, and 36.1% (276/764) had the codon TAC. Both TAC and TAT encode Tyr (Y) at the protein level (complete alignment can be found in Table S3, Supporting Information). Interestingly, the genomic contexts are highly conserved surrounding the RNA editing site in all 74 strains of *K. pneumoniae*, and all of them encoded TAC; furthermore, their motifs are in complete GTACGA (Figure 4C), suggesting the functional importance of this site on *badR* in *K. pneumoniae* which requiring maintains of local sequence motif for TadA recognition. Moreover, analysis of 563 publicly available RNA‐Seq datasets of *K. pneumoniae* from SRA (Table S4, Supporting Information) revealed 397 samples with at least ten reads covering position 3 969 107 on *badR*, and we detected editing in 309 samples (77.83%, Figure 4D). With the increasing coverage depth, we observed editing in a larger proportion of samples, which is up to 96.55% (Table S5, Supporting Information). Such strong conservation suggests that RNA editing may play an important role in *K. pneumoniae*.

## 3 Discussion

*K. pneumoniae*, a very important pathogen in the clinic, can cause a wide range of infections. To explore whether RNA editing regulation exists in *K. pneumoniae* and its biological functions, we identified RNA editing events by combining PacBio Sequel, Illumina WGS, and RNA‐Seq. As a result, we found a total of 13 RNA editing events, and all sites in coding regions recoded A‐to‐G mutations and resulted in Tyr‐to‐Cys transitions. The RNA loop structure conformed around most edited sites, and the sequence motif satisfied the binding structure required by TadA. Importantly, we found that RNA editing events on *badR* showed significantly different editing levels between hypervirulent and classic strains. The CRISPR/Cas9 system confirmed its function in the cell growth and virulence of *K. pneumoniae*. Furthermore, the strong conservation of sequence motifs and editing events on *badR* across *K. pneumoniae* strains indicated its importance during evolution.

The number of RNA editing events identified in this study is consistent with the number reported previously.^[^ ^12^, ^13^ ^]^ Several characteristics of the identified sites, along with the results of Sanger sequencing, confirmed that they are bona fide editing sites. Most RNA events occurred at wild or relatively low editing levels, which is similar to what has been reported in mammals.^[^ ^19^ ^]^ RNA editing sites are mainly located in noncoding regions in mammals; however, events in bacteria are mainly concentrated in coding regions, which might suggest that RNA editing in bacteria has a more direct effect than that in mammals. When we tried to perform GO enrichment analysis for genes under editing regulation, we failed due to the small number of genes; thus, we applied only GO annotations for these genes. By comparing RNA editing profiles between two *K. pneumoniae* strains with different virulence, we found that four editing events occurred at significantly different levels, while the others showed similar profiles.

We further focused on the RNA editing of BadR, which contains MarR‐type HTH domains that were reported to be transcription regulators of the MarR/SlyA family and are involved in the development of antibiotic resistance and virulence.^[^ ^26^, ^27^ ^]^ To evaluate the effect of the RNA editing of *badR* (A3969107G, Y99C) on *K. pneumoniae*, we simulated the effect of 100% editing on cell transcriptional maps by mutating the genome of the classic strain QD110. Based on RNA‐Seq analysis, a series of cellular quorum sensing genes (*lsrA*, *lsrB*, *lsrC*, *lsrD*, *lsrF*, *lsrG*, and *lsrK*) were downregulated in the mutant strain. These Lsr family genes are involved in AI‐2 importation and phosphorylation, which are associated with the intracellular signal transduction of stress responses such as biofilm formation and drug resistance;^[^ ^29^ ^]^ this result suggests that the RNA editing event in *badR* may affect intracellular signal transduction and cellular density changes. The wild‐type strain QD110 reached a higher limit in the cellular population than the mutant strain, accompanied by a decrease in the RNA editing level on *badR* (Figure S4, Supporting Information). This verified the dynamic regulation of RNA editing on *badR* according to cellular density and environmental nutrient deficiency. The regulation of the RNA editing level might help *K. pneumoniae* cells adapt to tough nutritional conditions by inducing intracellular communication factor production since one of the characteristics of reaching the culture stationary phase is a lack of nutrients. All these results suggest that RNA editing of *badR* may play role in the response to the *K. pneumoniae* population density of the bacterial culture. Reduced RNA editing is beneficial for the Isr family to promote systemic circulation of AI‐2. In turn, *K. pneumoniae* might better adapt to extreme environments by regulating intercellular communication. Mutant strains lack cellular communication factors when they face tough conditions, and this may be responsible for the low upper limit of cellular density during the stationary growth phase. Moreover, it has been reported that AI molecules participate in the regulation of virulence genes and the pathogenicity of gram‐negative bacteria.^[^ ^31^ ^]^ Similarly, the mutant strain presented a lower virulence than the wild‐type strain in the *G. mellonella* infection model, which may be attributed to the high RNA editing level of *badR*, consistent with the virulence difference between QD110 and R16. Here, we draw a brief illustration to describe the roles of RNA editing on *badR* in *K. pneumoniae* (Figure S7, Supporting Information).

Although we constructed a 100% editing strain to demonstrate the biological function of RNA editing on *badR*, it would be more convincing if we had a mutant system of the non‐editable codon. However, we tried many times, but none of them succeeded. In detail, a two‐plasmid pCasKP‐pSGKP system was employed to create the point mutation of the *badR* noneditable codon (TAC to TAT, Y99Y), which had previously been used to create the *badR* 100% edited mutation system (TAC to TGC, Y99C). We carried out ten mutation experiments using seven different complementary sgRNA sequences including the sgRNA that was used to successfully build the *badR*‐Y99C strain (sequences are listed in Table S6, Supporting Information). A total of 53 colonies grown on LB plates with two antibiotics were identified. PCR and Sanger sequencing were used to determine whether the point mutation was successfully created. However, all colonies remained wild type (TAC), except one that lost the targeting fragment (Figure S8, Supporting Information). Overall, we did not construct completely noneditable *badR* by gene editing. We then raised the question of whether this is because RNA editing regulation is very useful and under selective constraints in *K. pneumoniae*, which would lead to this site keeping the TAC codon all the time. Multiple sequence alignment of BadR orthologs from 66 bacterial species suggests interplay between the TAT and TAC codons at position 99 (Figure 4A,B) across different species. However, all 74 strains of *K. pneumoniae* encoded the motifs of GTACGA surrounding the RNA editing site (Figure 4C). Further analysis of public RNA‐Seq data of *K. pneumoniae* also confirmed that the RNA editing of *badR* occurred in most samples that we tested (Figure 4D and Table S5, Supporting Information). Such strong conservation of local sequence motifs and RNA editing events across different strains provides insight that the RNA editing of *badR* might play important roles in *K. pneumoniae*. These results might explain in part why the mutation system with completely noneditable codons was always unsuccessful. We suspect that noneditable codons may be lethal to bacterial cells.

## 4 Conclusion

In this study, through the analysis of the RNA editing regulation in hypervirulent and classic *K. pneumonia* strains, we reported that RNA editing has a certain regulatory effect on the growth and virulence of bacteria. Our conclusion suggested that in addition to mutation of the genome, it is necessary to consider regulation on the RNA level in future research on bacteria.

## 5 Experimental Section

### Bacterial Strains

The *K. pneumoniae* isolate R16 was collected from the drainage fluid for the liver abscess of a 26‐year‐old Chinese male patient with pseudocyst of pancreas infection. The *K. pneumoniae* isolate QD110 was collected from the blood of a 45‐year‐old Chinese female patient with a liver abscess. Isolates were sent to the central clinical microbiology laboratory of Peking Union Medical College Hospital (PUMCH) for identification confirmation using MALDI‐TOF MS (Vitek MS, BioMérieux, France). *G. mellonella* survival assays and human neutrophil assays were used for the virulence assessment of R16 and QD110.^[^ ^18^ ^]^

### RNA and DNA Extraction and Purification

RNA and DNA of the R16 and QD110 strains were purified using the GeneJET RNA Purification kit (Thermo Fisher Scientific) and Wizard Genomic DNA Purification kit (Promega), respectively, according to the manufacturer's protocol. Cultures were grown on LB supplemented with ampicillin (100 µg mL^−1^) at 37 °C. RNA and DNA were isolated from cultures at the middle of the logarithmic phase (OD600 in a 1‐cm cuvette of ≈0.6–0.8) for transcriptome sequencing and genome sequencing. Four replicates were performed for the identification of RNA editing.

### Library Preparation, Sequencing, and Quality Control for WGS, RNA‐Seq, and PacBio‐seq

For the whole genome‐seq, a total amount of 1.5 µg DNA per sample was used as input material for the DNA sample preparations. Sequencing libraries were generated using the Truseq Nano DNA HT Sample Preparation Kit (Illumina USA) following the manufacturer's recommendations, and index codes were added to attribute sequences to each sample amplification. Finally, PCR products were purified (AMPure XP system), and libraries were analyzed for size distribution by an Agilent2100 Bioanalyzer and quantified using real‐time PCR. For RNA‐seq, a total amount of 2 µg RNA was first treated with a Ribo‐zero rRNA removal kit (Illumina, USA). Subsequently, sequencing libraries were generated using rRNA‐depleted RNA with a VAHTS Strand‐specific mRNA‐seq Library Prep Kit with dUTP‐mode for Illumina (Vazyme, China) following the manufacturer's recommendations. The DNA and RNA libraries constructed above were sequenced by the Illumina Nova Seq platform, and 150 bp paired‐end reads were generated with an insert size of ≈350 bp. PacBio Sequel‐seq was completed in the previous work.^[^ ^18^ ^]^

### Sequence Alignment and Strategy for RNA Editing Identification

De novo assembly of the genome was performed using HGAP3 within SMRT Link v5.0.0 with PacBio sequencing data. Gap closing was completed by PBJelly,^[^ ^2^ ^]^ and circularization was achieved by manual comparison and removal of regions of overlap. The final genome was further confirmed by remapping Illumina reads using BWA (0.5.9) and Pilon (v.1.13). This assembled genome was published in the previous work.^[^ ^18^ ^]^ Then, RNA‐seq reads were aligned to this reference genome by BWA with default parameters, from which only uniquely mapped reads were retained. In addition, potential duplications were removed using Picard MarkDuplicates (2.18.7). Uniquely mapped RNA‐seq reads were then divided into two groups according to their Flag information in SAM format: reads transcribed from the plus strand and those from the minus strand. Single‐nucleotide variation (SNV) calling was separately performed for the two groups of reads using Samtools mpileup (v0.1.16). Reads harboring SNVs within 5 bp of both ends were discarded in SNV calling due to read‐end‐biased sequencing errors. A candidate RNA‐editing site was required to be homozygous, with 95% of the covered reads supporting the major allele type (DNA filter). RNA SNVs with a homozygous genotype were included in an initial list of RNA‐editing sites and were further subjected to a stringent filtering protocol: i) At least five RNA‐Seq reads with ≥2 nucleotides sequenced with high PHRED base quality (≥25) were required to support the variant form. ii) SNVs displaying more than one mismatch type were discarded. iii) For candidate RNA‐editing sites, BLAT alignment filtering was performed to eliminate SNVs potentially caused by misalignment to paralogs or pseudogenes; iv) A Strand Bias Filter, RNA‐editing sites exhibiting strand bias in read distribution (Fisher's exact test, *p*‐value< 0.05) or supported by <2 reads on either of the two strands were excluded.^[^ ^19^ ^]^ All editing sites were finally validated by Sanger sequencing with the primers listed in Table S6, Supporting Information.

### RNA Secondary Structure Prediction and Motif Analysis

To examine the RNA secondary structure, RNA sequences of 35 bases upstream and downstream from the edited site were extracted. Sequences were then entered into the RNAfold website (http://rna.tbi.univie.ac.at/cgi‐bin/RNAWebSuite/RNAfold.cgi) to predict the RNA secondary structure with minimum free energy. To further demonstrate the local sequence motif for TadA recognition, the surrounding sequences of editing sites were put into MEME (https://meme‐suite.org/meme/tools/meme) to identify the conserved motif.

### Protein Modeling for BadR

SWISS‐MODEL^[^ ^32^ ^]^ was used to generate a putative 3D model of wild‐type BadR and Y99C mutant using the crystal structure model of the transcriptional regulator NadR (PDB ID code: 5aiq). The model 5aiq had the highest sequence identity (52.59%) to BadR among the MarR family members whose apo crystal structure has been released on RCSB PDB. Also, this model had a relatively high GMQE score (0.75, top4). Sequence alignment was performed for BadR and 5aiq (Figure S9, Supporting Information). The generated structures were visualized using the PyMol program (DeLano Scientific).

### The Time Course Experiment of RNA Editing on *badR*

QD110 was cultured in an LB medium from an optical density of 0.02 at 600 nm by a shaker culture system. Growth kinetics assays were performed at 2, 4, 6, 8, and 24 h. RNA was isolated from cultures for RNA editing level measurements at sequential time points. One microgram of total RNA was reverse‐transcribed into cDNA for Sanger sequencing (Figure S10, Supporting Information) and RNA sequencing using the GoScript Reverse Transcription System (Promega). RNA editing levels were measured with RNA‐seq data. The cellular intensity was detected using a microplate reader (Synergy/LX, Biotek).

### Plasmid Construction and Transformation

As an unavailable screening drug for the multidrug‐resistant R16 strain, the mutation of A3969107G on *badR* and A5325069G on *virF* was performed in QD110 using a two‐plasmid *pCasKP*‐*pSGKP* system.^[^ ^33^ ^]^ The 20 nt complementary sequence of sgRNA was designed using the CRISPR guide RNA Design Tool (http://grna.ctegd.uga.edu/) and cloned into *pSGKP‐spe* between BsaI restriction enzyme cutting site using the Gibson Assembly kit (NEB, M5511A) *pBadRSGKP‐spe*. Repair template sequences were amplified and overlapped using PrimeSTAR Max DNA Polymerase (TAKARA). The primers are listed in Table S6, Supporting Information. Plasmids and PCR products were purified using a Plasmid mini kit (Omega) and Cycle pure kit (Omega) and concentrated using FreeZone TRIAD (LABCONCO) before the transformation. 200 nanograms of *pCas9KP‐apr* were transformed into QD110 competent cells using a Gene Pulser Xcell system (Bio‐Rad), and colonies were selected with 50 µg mL^−1^ apramycin. Then, 200 ng *pBadRSGKP‐spe* and 2 µg concentrated repair template sequence were transformed into QD110‐*pCas9KP* competent cells. Colonies were selected with 100 µg mL^−1^ spectinomycin and identified by Sanger sequencing (Figure S2, Supporting Information).

### Differential Gene Expression and Functional Annotation

To identify DEGs between WT and mutant strains, strand‐specific RNA‐seq was performed with three replicates for each strain. Differential expression analysis was completed using the DESeq2 package (version: 1.24.0) in *R*. The threshold for screening DEGs was | log2 FC (fold change) | > 1 and FDR < 0. 05. Enrichment analysis of KEGG was performed on DEGs using the clusterprofiler package (version: 3.18.1) treating expressed genes (RPKM >1) in any samples in WT and mutant strains under the *K. pneumoniae* (NTUH‐K2044, serotype K1) as background annotations. The significance of KEGG signaling pathways was set at a *p*‐value <0.05.

### Analysis of Autoinducer‐2 Production

*Vibrio harveyi* strain BB170 luminescence was introduced to calculate AI‐2 activity as described in previous research.^[^ ^34^ ^]^ *K. pneumoniae* WT and mutant strain badR‐99Cys were incubated in AB medium to induce AI‐2 production for 0, 2, 3, and 4 h at 37 °C, respectively. *K. pneumoniae* cells were removed by passing through 0.2 µm syringe filters. BB170 cells diluted in AB medium were co‐cultured with *K. pneumoniae* cell‐free cultures for a mixture ratio of 9/1 (v/v) at 30 °C for 6 h to calculate AI‐2 activity. The light production at a wavelength of 490 nm of BB170 was analyzed at the 3rd hour when the lowest bioluminescence was produced by the control. The induction of bioluminescence was calculated as the light output of the sample divided by the light output of the control.

### Cellular Growth Curve

The wild‐type strain and mutant strain *badR*‐99Cys were cultured at rest in 96‐well plates at an OD600 nm of 0.02 (biological replicates = 12). The experiments were repeated three times. The optical density was measured by the microplate reader (Synergy/LX, Biotek) every hour from 0 to 28 h. The cellular growth curve was drawn using GraphPad Prism software, and the Mann–Whitney test was used for comparisons between the wild‐type and mutant strains.

### *G. mellonella* Infection

Cells of the wild‐type strain and mutant strain *badR*‐99Cys were collected from cultures by centrifugation at 3000 rpm for 5 min. Then, the cells were washed twice and suspended at 10^8^ cells/mL in saline. Each *G. mellonella* was injected with a 10 µL suspension, with an infection dose of 10^6^ cells (*n* = 20). The death timepoint of each *G. mellonella* was observed at 8, 12, 15, 18, 24, 36, and 48 h after infection and there are 15 larvae for each group. Experiments were repeated three times. The survival curve of *G. mellonella* infection was drawn using GraphPad Prism software. The log‐rank (Mantel–Cox) test was used to compare survival rates between the wild‐type and mutant strains.

### Environmental Stress Stimulation

Variation of RNA editing level of *badR* was examined with stimulated by high temperatures, high salinity, and oxidation. QD110 strain was cultured in LB medium overnight and then diluted 100 times with LB broth containing nothing, 0.5 m KCl (high salinity), or 5 mm H_2_O_2_ (oxidation). All the strains were incubated at 37 °C, except high‐temperature stimulation at 48 °C, by a shaker culture system until reach the logarithmic growth phase (OD_600_ = 0.6–0.8). Three biological replicates were conducted for each condition and then RNA sequencing was applied to all samples.

### Analysis of *badR* Orthologs in Other Bacterial Species

To examine the amino acid composition at position 99 of badR, the BLAST tool on UniProt (https://www.uniprot.org/blast/) was used with the amino acid sequence of BadR in strain R16 as a query and “Microbial proteomes” as the target database. The alignment results were filtered with an e‐value of 1e‐5, identity of 80%, and coverage of 80%. A nonredundant set of orthologs with one BadR sequence per species was constructed. The corresponding nucleic acid sequences were downloaded from the European Nucleotide Archive (ENA) (https://www.ebi.ac.uk/ena/browser/home). Multiple sequence alignments were conducted with ClustalW.

### Phylogenetic Analyses

The 16S ribosomal RNAs were used to build a genus phylogenetic tree to visualize the amino acid composition in BadR's position 99 in an evolutionary context. The 16S ribosomal RNA from one representative from each genus (Table S7, Supporting Information) was used. Multiple sequence alignment was performed by using ClustalW (default parameters) embedded in the MEGAX package.^[^ ^35^ ^]^ The evolutionary tree was inferred by using the maximum likelihood method and the Tamura‐Nei model.^[^ ^36^ ^]^ The tree with the highest log likelihood (−4935.73) was shown. Initial tree(s) for the heuristic search were obtained automatically by applying the neighbor‐joining and BioNJ algorithms to a matrix of pairwise distances estimated using the Tamura‐Nei model and then selecting the topology with a superior log likelihood value. The tree was drawn to scale, with branch lengths measured in the number of substitutions per site (next to the branches). The proportion of sites where at least 1 unambiguous base was present in at least 1 sequence for each descendent clade was shown next to each internal node in the tree. This analysis involved 15 nucleotide sequences. There were a total of 1558 positions in the final dataset. Evolutionary analyses were conducted in MEGA X.

### Statistical Analysis

Statistical analyses were performed using the Statistical Package for Social Sciences. Data were presented as mean ± SD. Experiments were performed with a minimum of three replications. Mantel–Cox test, Mann–Whitney U test, Fisher's exact test, and Student's *t*‐test were used to identify significant differences where appropriate.

## Conflict of Interest

The authors declare no conflict of interest.

## Supporting information

Supporting Information

Supporting Information

## Data Availability

The data that support the findings of this study are openly available in Genome Sequence Archive (Genomic, Proteomics & Bioinformatics 2021) in National Genomics Data Center (Nucleic Acids Res 2022), China National Center for Bioinformation/Beijing Institute of Genomics, Chinese Academy of Sciences at https://ngdc.cncb.ac.cn/gsa, reference numbers CRA007948 and CRA010432.
