# Supplementary material for: A‐to‐I RNA Editing in Klebsiella pneumoniae Regulates Quorum Sensing and Affects Cell Growth and Virulence
Source: Adv Sci (Weinh). 2023 Apr 21;10(17):2206056. doi: 10.1002/advs.202206056 (PMC10265045; doi:10.1002/advs.202206056)
Supplement: Supplementary file 1 — Supporting Information [file ADVS-10-2206056-s001.pdf]

ADVANCED SCIENCE
Open Access

Supporting Information

for *Adv. Sci.*, DOI 10.1002/advs.202206056

A-to-I RNA Editing in *Klebsiella pneumoniae* Regulates Quorum Sensing and Affects Cell Growth and Virulence

Xin-Zhuang Yang, Tian-Shu Sun, Pei-Yao Jia, Sheng-Jie Li, Xiao-Gang Li, Yanan Shi, Xue Li, Haotian Gao, Huabing Yin, Xin-Miao Jia and Qiwen Yang**

Supporting Information

A-to-I RNA editing in Klebsiella pneumoniae regulates quorum sensing and affects cell growth and virulence

Xin-Zhuang Yang[#], Tian-Shu Sun[#], Peiyao Jia[#], Sheng-Jie Li[#], Xiao-Gang Li, Ya-nan Shi, Xue Li, Haotian Gao, Huabing Yin, Xin-Miao Jia[*], Qiwen Yang[*]

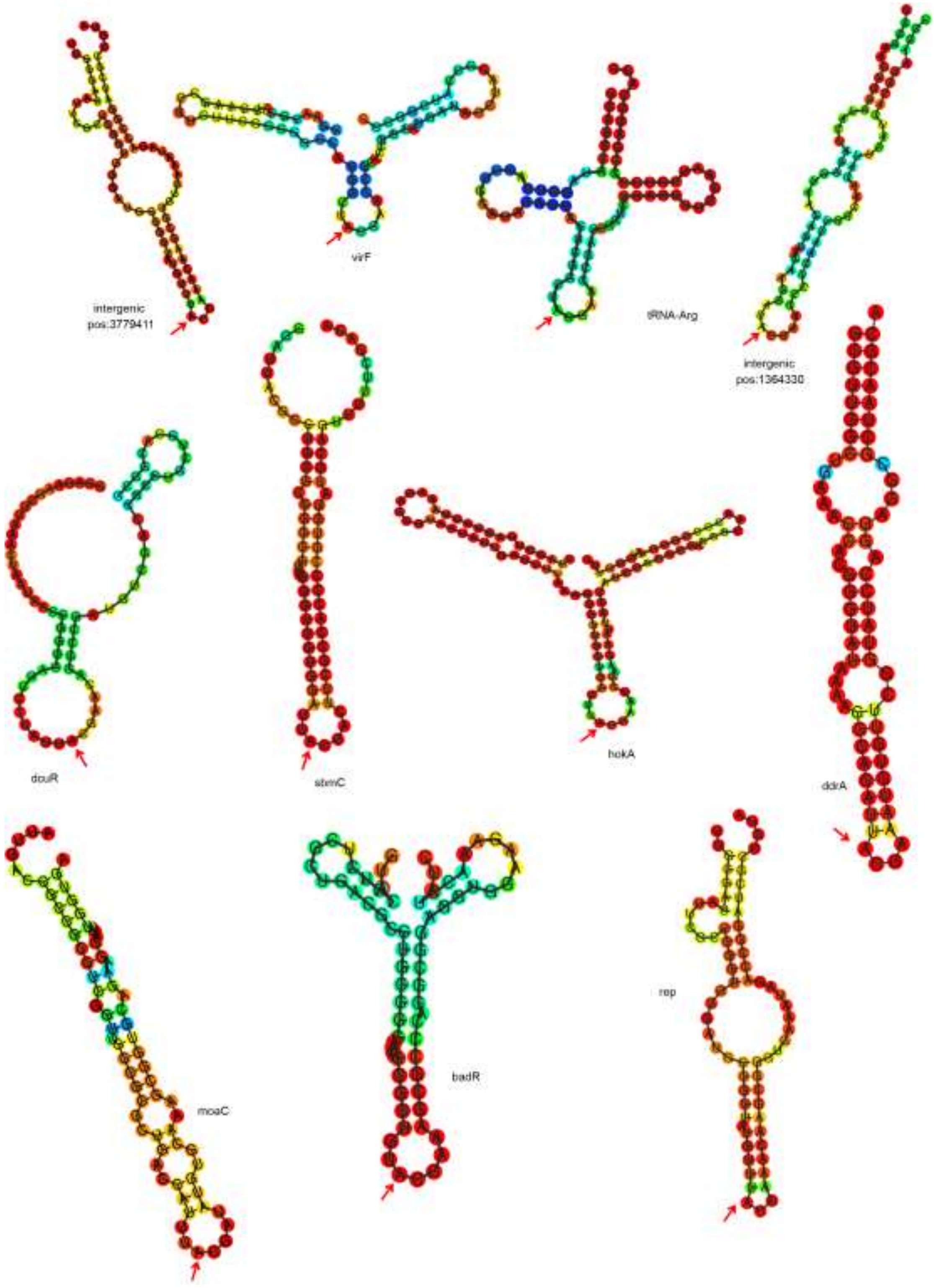

Figure S1. Predicted RNA secondary structure for all editing sites.

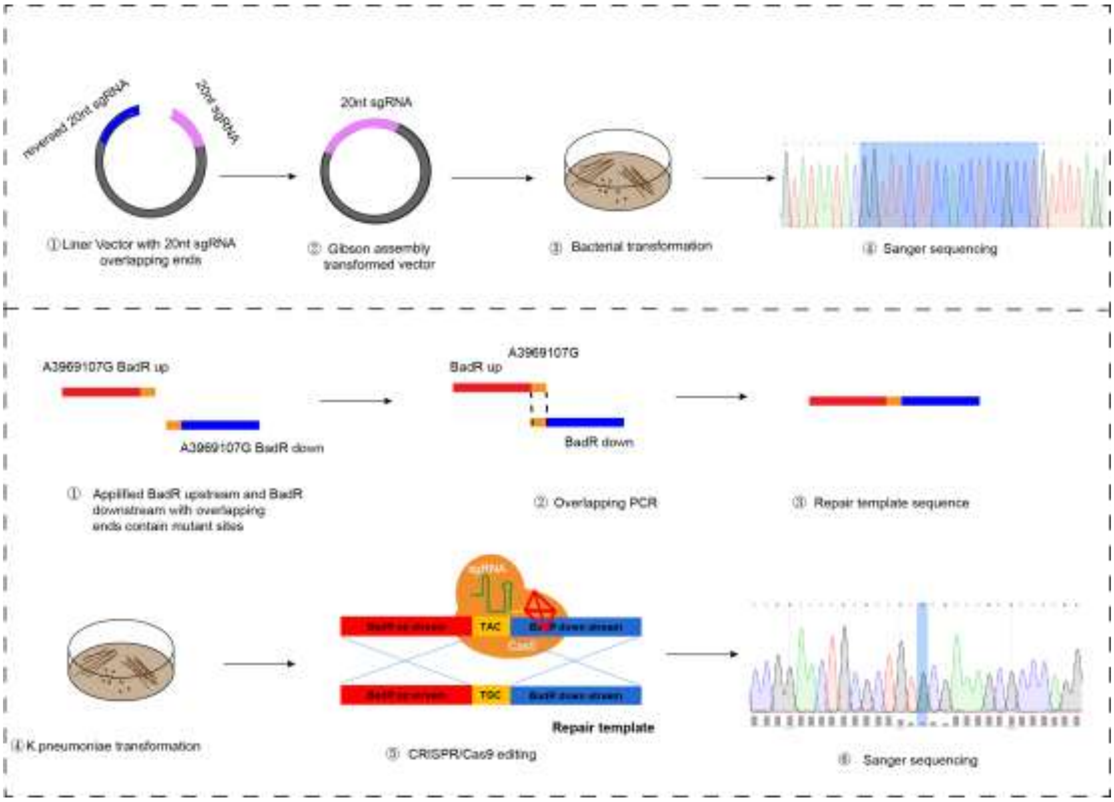

Figure S2. Construction process of the *badR*-99Cys mutant strain. The twenty-nt sgRNA sequence was coloured in a CRISPR/Cas9 engineered plasmid, which carried the Cas9 expression gene and sgRNA scaffold. The repair template of *badR*-99Cys (A3969107G) was amplified from the genomic DNA of QD110 with primers carrying the mutation site. Under the guidance of sgRNA, Cas9 cleaved the target gene. The target gene was mutated according to the repair template.

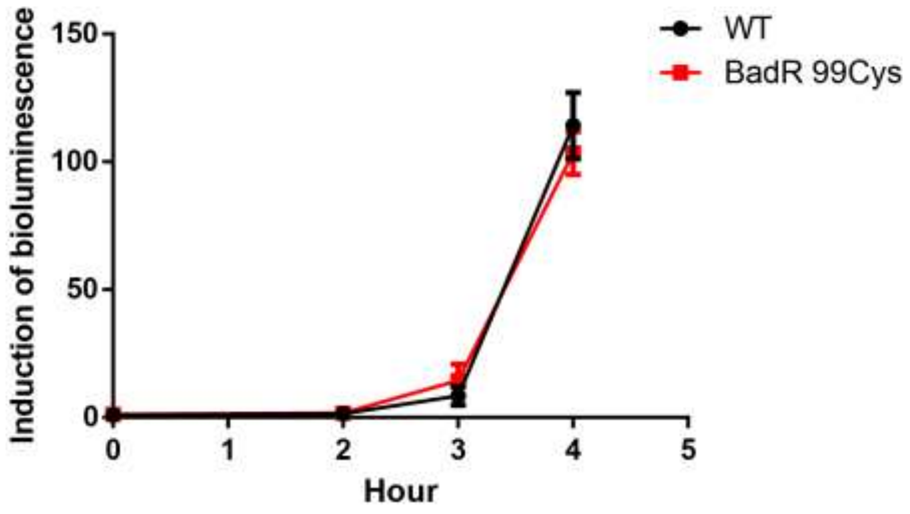

Figure S3. AI-2 production was dynamically examined in cell-free cultures incubated for 0~4 hours.

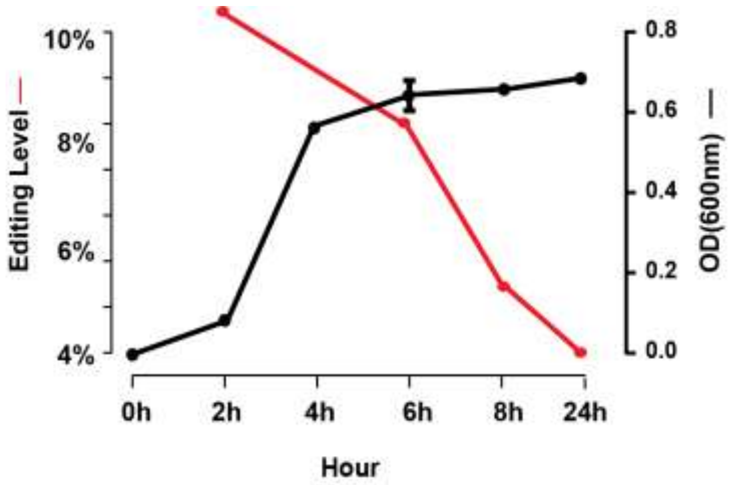

Figure S4. Change in the editing level of *badR* when cells grew from logarithmic phase to stationary phase.

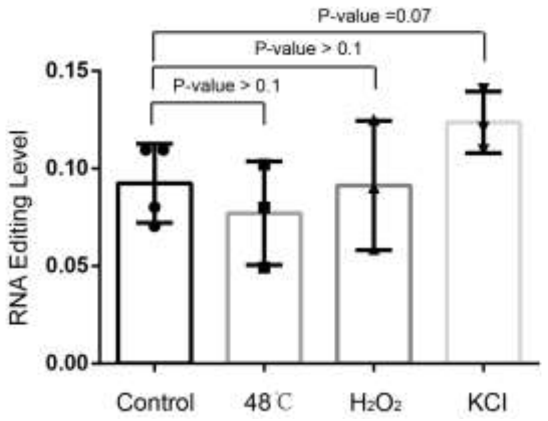

Figure S5. The changes in RNA editing levels of *badR* gene under different conditions of stimulation

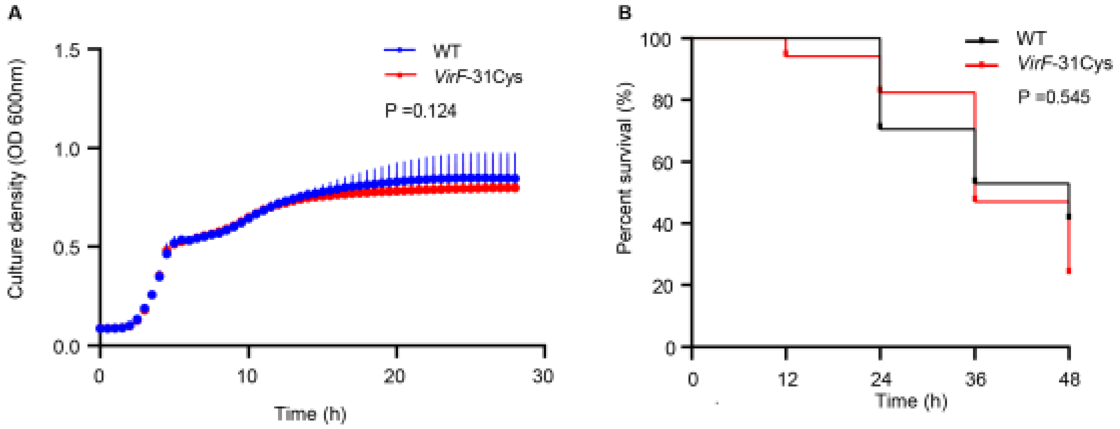

Figure S6. (A) The cellular growth rates of WT and *virF*-31Cys mutant strains were validated by recording the optical density at 600 nm of cell cultures for 48 hours. Each point represents the current cellular density each hour. There was no significant difference in growth rates between WT and *virF*-31Cys after the 8th hour (n=16; *P* value=0.124). (B) The virulence of WT and *virF*-31Cys was measured in the *Galleria mellonella* infection model with 15 larvae. The ordinate represents the percentage of surviving individuals at each time point. The endpoint was the 48th hour of infection. *virF*-31Cys showed a survival rate similar to that of WT after the 10th hour of infection (*P* value=0.545).

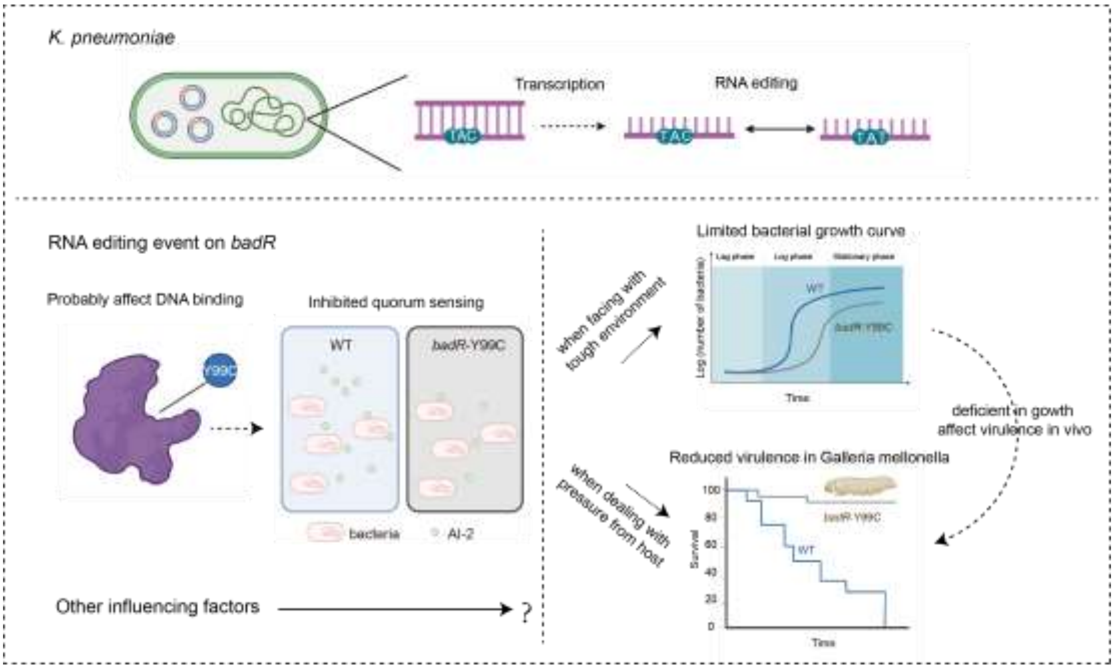

Figure S7. An illustration to describe the roles of RNA editing on *badR* in *K. pneumoniae.*

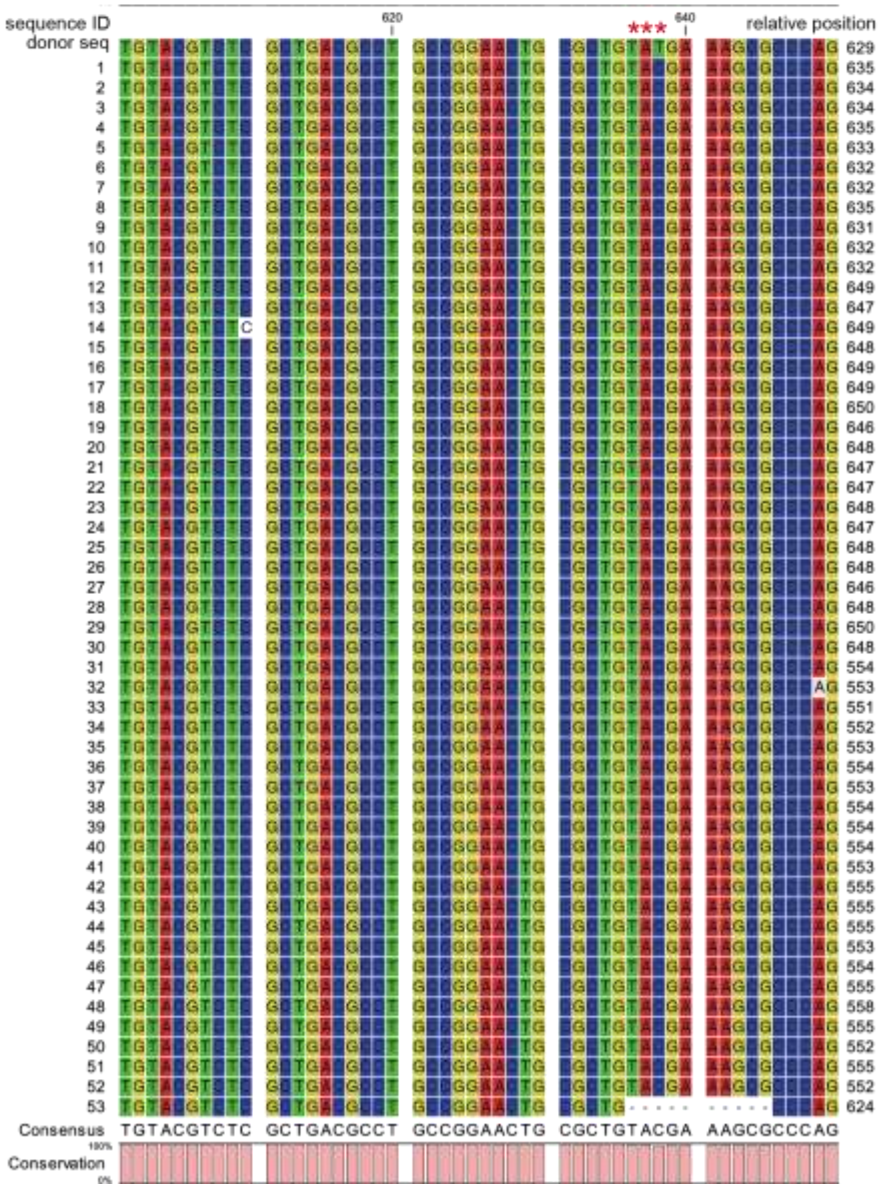

Figure S8. Alignment of the *badR* gene fragment containing the mutant site by Sanger sequencing. The codon is highlighted in red pentacles. TAC is the wild-type codon, and TAT is the unedited codon from the donor sequence constructed by fusion PCR.

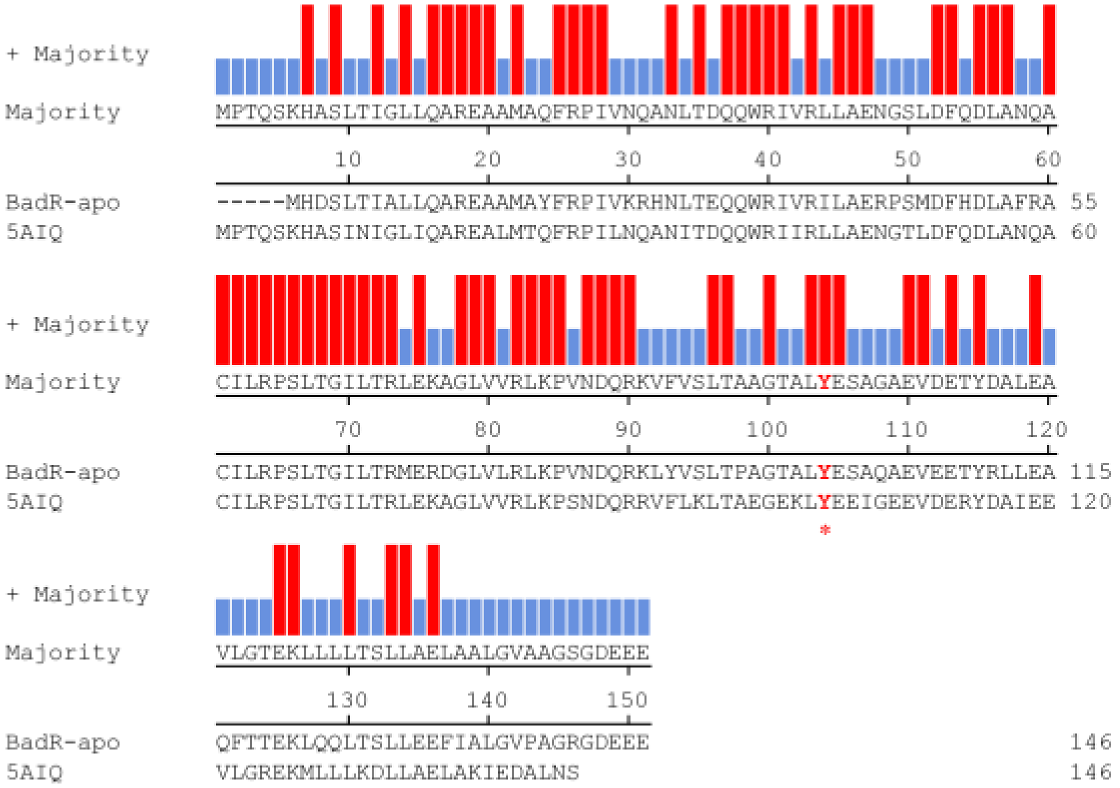

Figure S9. Sequence alignment for BadR and 5aiq.

2H (replicate 1~3)

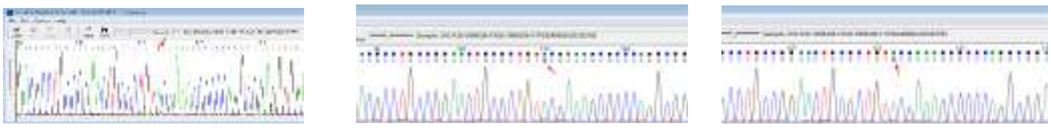

6H (replicate 1~3)

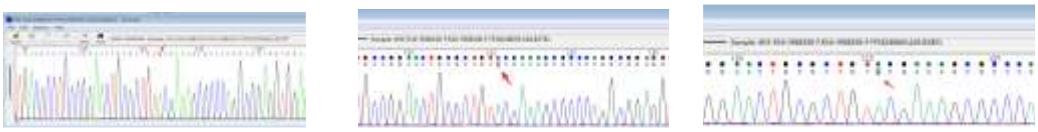

8H (replicate 1~3)

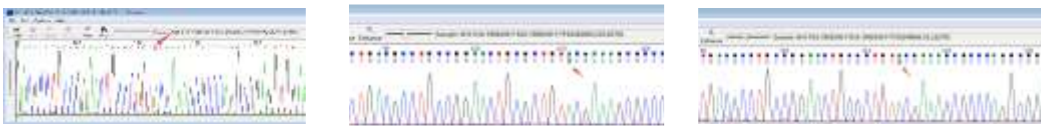

24H (replicate 1~3)

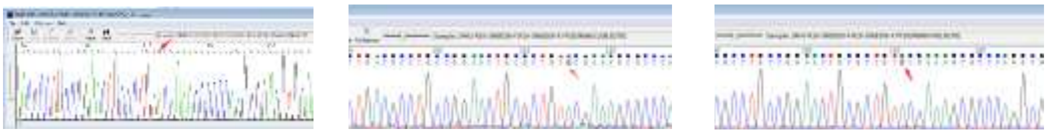

Figure S10. Sanger sequencing of RNA editing on *badR* over time.
