# Supplementary material for: A‐to‐I RNA Editing in Klebsiella pneumoniae Regulates Quorum Sensing and Affects Cell Growth and Virulence
Source: Adv Sci (Weinh). 2023 Apr 21;10(17):2206056. doi: 10.1002/advs.202206056 (PMC10265045; doi:10.1002/advs.202206056)
Supplement: Supplementary file 2 — Supporting Information [file ADVS-10-2206056-s002.zip › TableS5-S6.docx]

**Supplemental Table S5. Proportion of samples which detected RNA editing events on badR under different read coverage.**

| Depth cut-off | >10x | >20x | >30x | >50x | >100x |
| --- | --- | --- | --- | --- | --- |
| Total samples | 397 | 309 | 248 | 164 | 87 |
| Samples with editing | 309 | 267 | 218 | 153 | 84 |
| proportion | 77.83% | 86.41% | 87.90% | 93.29% | 96.55% |

**Supplemental Table. S6. All primers used in this study.**

| **Primer** | **Sequence** |
| --- | --- |
| *BadR* sgRNA-pSGKP F | GGTTTCTTCCACCTCCGCCTGTTTTAGAGCTAGAAATAGCAAGTTA |
| *BadR* sgRNA-pSGKP R | AGGCGGAGGTGGAAGAAACCGTATTATACCTAGGACTGAGCTA |
| *BadR* RT up F | TCTTTTATGCTTTGCGCTGA |
| *BadR* RT down R | CTGGCAGAAACTGATCCTCTG |
| *BadR*-Cys RT up R | CAGCGCAGTTCCGGCAG |
| *BadR*-Cys RT down F | CTGCCGGAACTGCGCTGTGCGAAAGCGCCCAGGCGGA |
| *VirF* sgRNA-pSGKP F | TAGTGCCGGTGCGGCTCATAGCCG |
| *VirF* sgRNA-pSGKP R | AAACCGGCTATGAGCCGCACCGGC |
| *VirF* RT up F | GGAGATGTTCGCCCAGATAG |
| *VirF* down R | GCTGATAAACTGGCGCGTCA |
| *VirF*-cys RT up R | GCCGTGGCCGCCGAAG |
| *VirF*-Cys down F | CTTCGGCGGCCACGGCTGCGAGCCGCACCGGCATG |
| *rpoB*-PCR-F | AGGATATGATCAACGCCAAG |
| *rpoB*-PCR-R | GGGTTGTTCTGGTCCATAAA |
| 4939002-F | TGAAATGCAGGCGACGACCC |
| 4939002-R | CGCTGGCCACTGGATGTTCG |
| 5325069-F | GGAATAACCCCTCAAGAACG |
| 5325069-R | GGAATAAACGGTAGTGGCCT |
| 368436-F | AGGAAACGCGGGAGATATTC |
| 368436-R | AGGCCCAGGAGATGATTAAC |
| 2151769-F | GATCTACCGTCTGAAAAGCC |
| 2151769-R | ACAATTCAGATGTTAGTCTC |
| 1393718-F | CATGTCGATATCCCAGTAGC |
| 1393718-R | GGATCACACCGTCAAGCAGG |
| 2255195-F | AACTCAAAGGCGACGATTAA |
| 2255195-R | TGATGGTTTCCGTGATGGTT |
| 3968107-F | CCTTTCGCGCCTGTATCCTG |
| 3968107-R | ACCGCTACTCTTCTTCATCG |
| 34294883-F | CCTTCCTGCGTATCGTCAAC |
| 34294883-R | CAGGGTAAAGCGGGTGACCA |
| tRNA-Arg-F | CGCAGCCTACGCAACAACAT |
| tRNA-Arg-R | ATTCTCCTTAGTAACAGCAA |
| 1364330-F | TCGGCAAACGCAGTATTACC |
| 1364330-R | TTGACATCCTTTGGTGCAGC |
| 3779411-F | AGTGATGTCCGAAGTCAATC |
| 3779411-R | CGTGGATATCGATAACTACC |
| sgRNA2-badR-F | TGCGCTGTACGAAAGCGCCC |
| sgRNA3-badR-F | GTACGAAAGCGCCCAGGCGG |
| sgRNA4-badR-F | CGAAAGCGCCCAGGCGGAGG |
| sgRNA5-badR-F | GCTGTACGAAAGCGCCCAGG |
| sgRNA6-badR-R | TCGTACAGCGCAGTTCCGGC |
| sgRNA7-badR-R | GCTTTCGTACAGCGCAGTTC |
